# Innovativeness of Industrial Processing Enterprises and Conjunctural Movement

**DOI:** 10.3390/e22101177

**Published:** 2020-10-19

**Authors:** Aleksander Jakimowicz, Daniel Rzeczkowski

**Affiliations:** 1 Department of World Economy, Institute of Economics, Polish Academy of Sciences, Palace of Culture and Science, 1 Defilad Sq., 00-901 Warsaw, Poland; 2 Department of Market and Consumption, Institute of Economics and Finance, Faculty of Economic Sciences, University of Warmia and Mazury in Olsztyn, 1/327 Cieszyński Sq., 10-720 Olsztyn, Poland; daniel.rzeczkowski@uwm.edu.pl

**Keywords:** macroeconomics, innovative activity, manufacturing industry, conjunctural movements, cybernetics, feedback loops, correspondence analysis, Polish Green Island effect, Red Queen effect, Kondratieff waves

## Abstract

Singulation of components determining the innovative activity of enterprises is a complex issue as it depends on both microeconomic and macroeconomic factors. The purpose of this article is to present the results of research on the impact of the mutual interactions between ownership and the size of companies on the achievement of the objectives of innovative activity by Polish industrial processing enterprises in changing cyclical conditions. The importance of innovation barriers was also assessed. Empirical data came from three periods that covered different phases of the business cycle: prosperity 2004–2006, global financial crisis 2008–2010, and recovery 2012–2014. The research used a cybernetic approach based on feedback loops presenting interactions between variables. In addition, two statistical methods were used: the Pearson’s $\chi^{2}$ independence test and correspondence analysis. The following discoveries were made during the research: (1) consideration of the combined impact of ownership and the size of companies on their innovation activities makes it possible to study phenomena that may be overlooked if the impact of these factors is considered separately; (2) public enterprises achieve significantly worse results in terms of innovation than companies from other ownership sectors; (3) the Red Queen effect, which assumes that the best innovative enterprises exert selection pressure on all other companies, applies to industrial processing companies, and in particular public enterprises; (4) the industrial processing section is more sensitive to secular trends than to cyclical fluctuations; (5) confirmation of occurrence of the Polish Green Island effect, which assumes that companies achieve good results in terms of innovation, irrespective of the phases of the business cycle; and (6) statistical evidence is provided that the global financial crisis may be associated with the turn of the Fifth and Sixth Kondratieff waves. Most likely, the role of the communication channel between the world economy and the Polish manufacturing section is fulfilled by foreign ownership, whose percentage of share capital of this section is estimated at 50%.

## 1. Introduction

The explanation of the relationship between innovation and conjunctural phenomena is one of the most important problems of modern economics. By their very nature, the processes that should be taken into account during such studies are long-term. Therefore, they should be considered not only within the time frame appropriate for traditionally understood business cycles, but also from the perspective of secular changes. At the heart of conjuncture theory (as well as of business cycle theory) is the division of the stunning complexity of economic interactions into a number of heterogeneous forms of movement according to the criterion of their duration. According to the guidelines of the Harvard Business School, we can distinguish the following forms of movements: (1) the fundamental course of movement (or secular trend), (2) seasonal fluctuations, (3) cyclical fluctuations (conjuncture in the narrower sense), and (4) miscellaneous random fluctuations. With this approach to the problem, business cycle theory tackles the cyclical movements in a narrower sense, and thus variations of economic phenomena that are recurring in free rhythm [1]. Conjunctural movements refer to the entire wavelike evolution of economic life, so by definition, they include secular changes, which is well documented in the literature [2,3,4,5,6]. This is the justification for the use of the mentioned term in the title of the article, as we believe that it is advisable to extend the time frame of studies on innovation beyond those that are appropriate for traditionally understood business cycles.

It is difficult to imagine an increase in the innovative activity of enterprises without prior changes in production techniques, which usually occur over long periods. This implies the need to study interdependencies between innovation and traditional business cycles and secular cycles. Technological revolutions belong to the secular factors of economic growth and development and are the cause of supercycles or Kondratieff waves (K-waves), which last from 48 to 60 years. Changes occurring over several decades, as opposed to business cycles, are usually caused by extra-economic circumstances and events. According to Kondratieff, secular factors can be divided into the following four groups [7]: (1) changes in technology, (2) wars and revolutions, (3) the assimilation of new countries into the world economy, and (4) fluctuations in gold production. In the case of the Polish industrial processing section, at least two factors should be taken into account, the first and third, and maybe even all of them.

The modification of the Kondratieff long-waves theory was made by Šmihula, according to whom the global financial crisis is a phenomenon typical of the breakthrough associated with the end of one and the beginning of the next K-wave [8]. In this case, it would be a transition from the Fifth to Sixth K-Wave, which would mean the end of the information and telecommunications revolution and the initiation of the biomedical-hydrogen revolution. According to Šmihula, this breakthrough dates to 2015. The technological innovations underlying the secular cycles are usually the result of earlier technological revolutions. In addition, all long-waves have common features that are the cause of certain patterns of economic development. Each of them consists of two phases: the innovation phase in which the inventions find practical applications, and the application phase in which the existing innovative solutions are improved and integrated into everyday economic life. The end of a given wave of innovations is determined by the decreasing rate of profit from a new innovation to the level appropriate for traditional branches of industry. Thereby, a given technology achieves its proper development limit. In order to cross the limit, a new innovative technology is required. Thus, the end of the application phase of each K-wave signifies a period of stagnation caused by the economic crisis, which can be overcome by increased demand for new inventions and revolutionary technological innovations. In addition, Šmihula made an interesting observation regarding the shortening of the length of successive K-waves [8]. This implies the constant acceleration of technological development, which—if this trend continues—may lead in the years 2080–2090 to blurring of the differences between K-waves and classical business cycles.

Schumpeter’s theory of innovation, which has been elaborated in the first half of the 20th century is the milestone of the study of interdependencies between innovation and economic growth and development. Schumpeter perceived innovation as the driving force of the economy, which on one hand ensures its development, but on the other hand, is the source of the business cycle because it brings the economy out of balance. In his opinion, innovations are at the center of almost all socio-economic phenomena, and the length of the two basic phases of the business cycle, prosperity and recession, depend on the essential features of the innovation that underlie the cycle [9]. The main figure in his theory is the entrepreneur, whose basic task is to search for new combinations of productive means, therefore, such that were not created as a result of improving existing combinations. These include the following five cases [10]: (1) placement of new products on the market, (2) implementation of new production methods, (3) opening of new markets, (4) acquisition of a new source of raw materials or semi-manufactured goods, and (5) introduction of a new organization of any industry.

Schumpeter’s interests also included issues related to the impact of ownership and size of enterprises on their innovation activity. The essence of ownership is the ability to freely dispose of means of production, which can be used directly to create a new combination of forces and materials or can be exchanged for the necessary goods and services. In his opinion, during the capitalist process, dematerialization of property occurs, as a result of which property ceases to perform its basic functions in business. Elimination of the material substance of property, which is done by exchanging factory walls and machinery for a mere parcel of shares, deprives ownership of its most important feature, which is moral allegiance. In this way, the holders of the title cannot freely dispose of their property. With respect to the size of the company, it is very important for innovation. In a competitive economy, new enterprises are the carriers of innovation, and these companies are not necessarily large. The situation is changed by the emergence of huge concerns that reduce the competitiveness of the economy and gain an advantage in the field of innovation over smaller companies due to their size [10,11]. The theory of innovation presented here shows that ownership and size are the basic factors determining the innovativeness of enterprises, therefore their impact must be considered together. Thus, innovations and related economic phenomena should be viewed from the point of view of cybernetics, where the importance of feedback loops is emphasized.

The aim of the article is to complete the gap in research on innovation, which consists of the failure to define the interrelationships between the innovative activity of enterprises at the microeconomic level and long waves occurring at the macroeconomic level. Most studies have focused on the separate impact of variables such as the type of enterprise and ownership sector on innovation activity and barriers to innovation, however, it is very important to capture the combined impact of these variables. The article proves that consideration of the combined impact of the type and ownership sector of enterprises on their innovative activity allows for the discovery of previously unknown economic phenomena. It should be emphasized that the adopted research methodology is characteristic of the complexity economics and therefore the macroeconomic and microeconomic levels are not distinguished [12] (pp. 97, 161–185). This allowed for the following discoveries: (1) consideration of the combined impact of ownership and the size of companies on their innovation activities makes it possible to study phenomena that may be overlooked if the impact of these factors is examined separately; (2) public enterprises achieve significantly worse results in terms of innovation than companies from other ownership sectors; (3) the Red Queen effect, which assumes that the best innovative enterprises exert selection pressure on all other companies, applies to industrial processing companies, and in particular public enterprises; (4) the manufacturing sector is more sensitive to secular trends than to cyclical fluctuations; (5) confirmation of occurrence of the Polish Green Island effect, which assumes that companies achieve good results in terms of innovation, irrespective of the phases of the business cycle; and (6) statistical evidence is provided that the global financial crisis may be associated with the turn of the Fifth and Sixth Kondratieff waves.

As noted by W. Brian Arthur [13] (pp. 16–17), the source of the complexity of economic systems is the presence of both negative and positive feedbacks, the effects of which overlap. The feedbacks from the manufacturing sector are presented below in cybernetic diagrams (2)–(8), which are used to study the mutual interactions between: ownership sector and enterprise type, ownership sector + enterprise type and innovative activity, ownership sector + enterprise type and barriers to innovation, barriers to innovation and innovative activity, and innovative activities in different periods. In many cases, the strength of the feedbacks is examined on a four-point scale: high, medium, low, and irrelevant. It is observed that—depending on specific conditions—all these feedbacks can be both positive and negative, and their effects overlap, which creates a complex pattern of industrial processing in Poland. The innovative activity of enterprises also depends on many other factors such as the national and international environment [14,15,16], management system [17], business support organizations [18,19], intellectual assets [20], sectoral patterns of cooperation and technology level [21], for which the *ceteris paribus* assumption was made. It can also affect other economic variables such as total factor productivity [22], the level of firm productivity [23], and anti-crisis reputational sustainability [24]. These dependencies create additional feedback loops that spread throughout the economy and increase its complexity.

In the manufacturing sector in Poland, there are also at least four other sources of complex dynamics, apart from the positive and negative feedbacks discussed above. First, the innovative activity of enterprises is more dependent on secular factors than on the phases of the business cycle. Another big surprise was the steady decline in the significance of innovation barriers in successive periods prosperity (2004–2006), global financial crisis (2008–2010), and recovery (2012–2014). This brings to mind the fractal market hypothesis, which applies to capital markets, and highlights the importance of time scales in which investors operate [25]. Second, the industrial processing sector can be considered as a complex adaptive system in the meaning of Gell-Mann [26], because the operation of its companies is based on the creation and improvement of schemas or models describing the regularities observed in the environment. These schemas are then used by companies to operate in the real world. Third, the sector under study can be viewed from the point of view of thermoeconomics and trends analyzed as a result of changes in thermodynamic entropy and money entropy [27]. Fourth, entropy and information are closely related, leading to the conclusion that entropy can be used in economics to measure ignorance or uncertainty. Information and ignorance are opposites, but the measurement of one quantity can determine the other [28]. Ignorance related to thermodynamic entropy applies to both innovative strategies of industrial processing enterprises and the government’s pro-innovation policy, therefore it is both microeconomic and macroeconomic in nature. Ignorance related to money entropy concerns the disorder of monetary policy, so it is only macroeconomic in nature.

## 2. Materials and Methods

### 2.1. General Characteristics of the Cybernetic Research Approach

Contemporary studies on innovation are dominated by the concept that ownership and the type of enterprise are treated as one of the most important variables determining the innovative activity of enterprises [29,30,31,32]. However, the impact of these factors is relatively often considered separately. This reasoning can be represented using mathematical formalism in the form of the following logical dependence:(1)
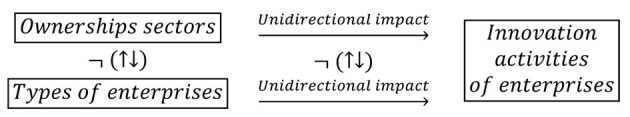


Negation symbol preceding two opposite arrows in brackets ¬ (↑↓) signifies bypassing the interactions between the ownership and size of the company, so each of these variables affects the innovation of companies separately. Furthermore, relationships between independent variables and the dependent variable are unidirectional. Feedbacks between innovation activities and the cumulative interaction of ownership and company size are therefore not included. Elimination of interactions between the ownership sector and the type of enterprise may critically affect the obtained results. Some publications recognize this problem [33,34].

This study used a cybernetic approach to the problem, which emphasizes the importance of feedback loops. After considering them, the logical relationship (1) is transformed into the following form:(2)
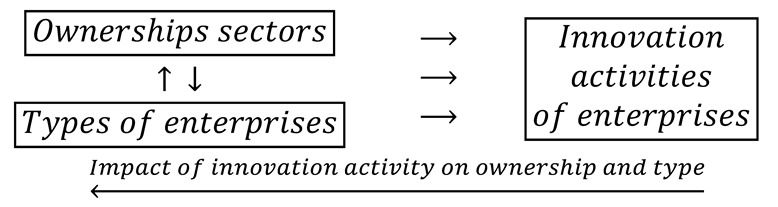


Such formulation of the problem allows for the examination of the impact of mutual interactions between ownership and type of enterprises on the innovation activity of enterprises. It also recognizes another, no less important feedback loop between variables containing a response record in the form of the impact of innovation activity on ownership and size of the enterprise. Therefore, the cybernetic scheme (2) is consistent with Schumpeter’s theory of innovation.

The cybernetic approach was aimed at simultaneous examination of the following phenomena: (1) determination of the mutual influence of ownership sectors and types of enterprises on the innovative activity of companies, (2) determination of the mutual impact of ownership sectors and types of enterprises on innovation barriers, and (3) registration of the impact of barriers to innovation on the innovative activity of enterprises. In addition, other feedback loops were included depending on the information contained in individual databases. Innovations implemented in a given period may contribute to the growth of innovations in subsequent years by affecting independent variables [35,36,37].

The research used three databases on Polish industrial processing enterprises, each of which covered one of the following periods: prosperity from 2004–2006, global financial crisis from 2008–2010, and recovery from 2012–2014. The first database contained 10,149 enterprises, the second included 20,655, and the third 10,244 (Table 1) [36]. The data were collected by the Statistical Office in Szczecin based on the PNT-02 questionnaires, which were subject to some modifications in the above-mentioned periods, but these changes were not significant enough to polarize observations in a way that could hinder the comparability of the research results. Nevertheless, there were some differences in the design of questionnaires in each of the periods, which as a consequence necessitated the development and adaptation of the relationship (2) to each of the three-year periods of analysis [35,36,37].

Three ownership sectors, public, private, and mixed (50% public, 50% private), and three types of enterprises distinguished on the basis of the size criterion (i.e., small, medium and large were considered in this study). The typology of enterprises is based on European Union standards, where certain thresholds are considered in the form of the number of employees and the volume of annual turnover or the annual balance sheet total (Table 2) [38].

The research adopted the following method for encoding variables. Small, medium, and large enterprises were indicated by symbols FR_1, FR_2, and FR_3, respectively, while ownership sectors —public, private, and mixed—were represented by the symbols S1, S2, and S3, respectively. The analyses concerning the combined impact of ownership sectors and types of enterprises on the objectives of innovative activity or barriers to innovation, which are presented in logical relationships (3)–(8), used two-part designations that first identified the ownership sector, and second, the type of enterprise. In this convention, medium-sized private sector enterprises were represented by the symbol S2FR_2.

Table 3, Table 4 and Table 5 contain the percentage data referring to types and ownership sectors of enterprises in the three periods under examination (i.e., 2004–2006, 2008–2010, and 2012–2014) [37]. Each of these tables shows both the share of particular types of enterprises in the ownership sectors and the share of particular ownership sectors in the types of enterprises. Table 3, Table 4 and Table 5 are to be read as follows. The Type column provides the percentage share of a given type of enterprise in individual ownership sectors. Table 3 shows that in the first period, medium-sized enterprises (FR_2) accounted for 4.14% of the public sector (S1), 83.64% of the private sector (S2), and 12.22% of the mixed sector (S3). The Subtotal (FR) row contains the percentage shares of each type of enterprise in the total number of enterprises. It indicates that in the prosperity period (2004–2006), small enterprises accounted for 30.06%, medium enterprises for 55.66%, and large enterprises for 14.28% of the total number of enterprises. With regard to the Sector column, it represents the share of a given ownership sector in each type of enterprise, which is read horizontally, taking into account every second cell of a given row. To clarify this, the Mixed (S3) row can be examined here. It demonstrates that the mixed sector comprised 44.84% of small enterprises, 41.87% of medium-sized enterprises, and 13.29% of large enterprises. The Subtotal (S) column shows the percentage of enterprises from the given ownership sector in the total number of enterprises. As can be inferred from Table 3, in 2004–2006, the public sector (S1) included 4.37%, the private sector (S2) was 79.39%, and the mixed sector (S3) was 16.24% of the total number of the investigated enterprises. Table 4 and Table 5 should be read in the same way. The data provided in Table 3, Table 4 and Table 5 relate to the role and importance of individual types and ownership sectors of enterprises in the whole section of industrial processing. They enable precise interpretation of correspondence maps showing the co-occurrence of points representing the types and ownership sectors of enterprises, points indicating the effects (objectives) of innovative activity, and points responsible for innovation barriers.

In this study, ownership sectors and types of enterprises are grouping variables. This results from the adoption of the Schumpeterian point of view, according to which these variables and the interactions between them exert a crucial influence on the effects and objectives of innovative activities undertaken by companies. In this way, the data were sorted into categories and groups with clear economic sense.

### 2.2. Feedback Loops in the Years of Prosperity

In the period of prosperity from 2004–2006, the impact of the mutual interactions of ownership sectors and types of enterprises on the effects of innovation activities and the degrees of influence of each of them on the activities of enterprises at the end of 2006 were determined. This problem can be illustrated by the following logical dependence:(3)
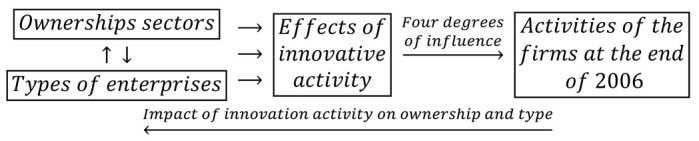


Relationship (3) contains an additional feedback loop between the effects of innovation activities in 2004–2006 and the activities of companies at the end of 2006. Four degrees of impact are possible: high, medium, low, and irrelevant. In addition, during the prosperity period, it was necessary to recognize the impact of interactions between ownership sectors and business types on barriers to innovation, as illustrated by another relationship:(4)
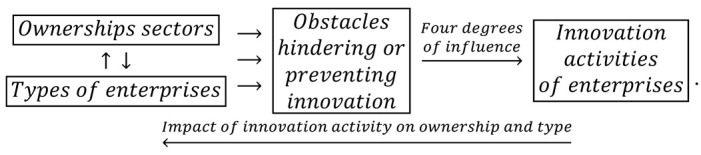


Innovation barriers may hinder enterprises from conducting innovative activities and even influence the decision not to conduct such activities. In this case, there are four degrees of the impact of innovation barriers on innovation activities: high, medium, low, and irrelevant. Undoubtedly, the considered feedbacks between innovation activities conducted at different times, as illustrated by relationship (3), and feedbacks between innovation activities and innovation barriers, as shown by relationship (4), are mediated by independent variables (i.e., ownership sectors and types of enterprises).

Nine effects of innovative activity and eleven barriers to innovation were taken into account during the period under study. Each of these variables can occur in four states, which, in conjunction with the nine states that ownership sectors and types of enterprises can collectively adopt, indicates the need to consider the simultaneous relationships between eighty-nine variable states.

### 2.3. Feedback Loops in the Years of the Global Financial Crisis

During the global financial crisis, there were slight changes in the statistical form PNT-02, which consisted of replacing effects with the goals of innovative activity and degrees of influence with degrees of importance. This time, the impact of the achieved goals on innovative activity in the field of product and process innovations in the years 2008–2010 was taken into account. The scale of impact included four degrees of importance: high, medium, low, and irrelevant. The overall cumulative impact of ownership sectors and types of enterprises on the goals of innovative activities and the degrees of their impact on the activities in the field of product and process innovation is presented in the cause and effect loops in the form of:(5)
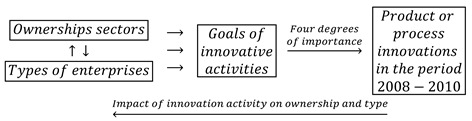


During this period, the impact of mutual interactions of ownership and enterprise type on innovation barriers and the four-level significance of these barriers for the innovative activity of companies were examined. Barriers to innovation may have impeded the conducting of innovation activities or influenced the decision not to conduct such activities. It is important to consider the impact of innovative activity on innovation barriers through the cumulative impact of ownership and types of enterprises. Therefore, the following logical relationship is addressed below:(6)
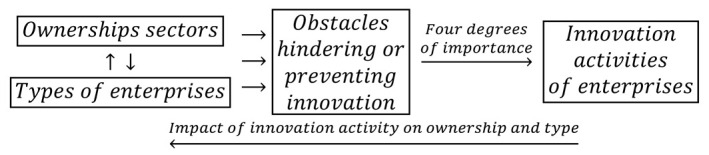


During the crisis years, the goals of innovative activities are described by ten variables and the barriers to innovation by eleven variables. All these variables can be in four states, which, together with nine states of ownership sectors and types of enterprises, indicates the necessity to examine interdependencies between ninety-three states of variables.

### 2.4. Feedback Loops during the Recovery Period 2012–2014

During the recovery period 2012–2014, the goals of the innovative activity of enterprises included four types of traditional innovations (product, process, organizational, and marketing innovations) and eco-innovations. During this period, the determination of the degrees of influence (importance) of these objectives on the further innovation activities of companies was abandoned. The feedback loops used in the research have the following form:(7)
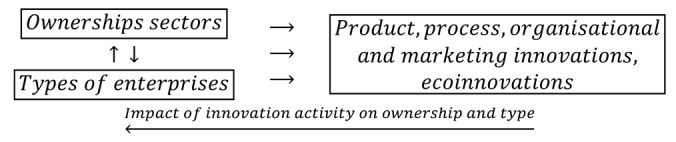


In the years of prosperity, as in the previous two periods, the joint impact of ownership sectors and types of enterprises on innovation barriers was also examined. The barriers to innovations included eleven variables, and their importance to the innovation activities of companies were on a four level scale. The barriers may have contributed to the lack of innovations to a high, medium, and low degree or be irrelevant. The study of the importance of innovation barriers for the innovative activities of enterprises was based on the following feedback loops:(8)
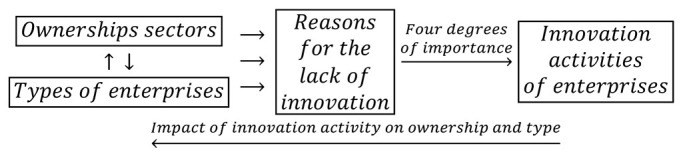


During this period, twenty-two goals of innovative activities were one-state variables, while eleven barriers to innovation were described by four-state variables. Considering nine ownership states and types of enterprises, it is needed to determine the interdependencies between seventy-five variable states.

### 2.5. Statistical Methods

Two statistical methods were used in the study: Pearson’s $\chi^{2}$ independence test and correspondence analysis. These methods were selected in such a way that the results obtained complement each other. The starting point for the calculations according to both methods is the summary of the data in the contingency tables. The first method tests the existence of significant relationships between variables, while the second provides information about the structure of the relationships between rows and columns of a contingency table.

The independence test is used to determine the relationship between two categorical variables [39]. The test relies on the comparison of the values resulting from (empirical) research with the expected values, which assume no relationship between variables. The options considered must be mutually exclusive and have a total probability of 1. The $\chi^{2}$ statistic is used to evaluate the test value. The choice between the null hypothesis on the independence of variables and its opposite (i.e., the alternative hypothesis) is made on the basis of a comparison of the *p*-value with the significance level.

Correspondence analysis is a multidimensional statistical method for studying co-occurrence of phenomena [40,41]. It has an exploratory character, which differs from traditional methods of testing statistical hypotheses. Classic methods rely on a priori verification of hypotheses regarding relationships between variables, while correspondence analysis enables the discovery of systematic relations between variables without formulating expectations a priori about the nature of these relationships. Therefore, correspondence analysis is not a confirmation technique, but a method of discovering relationships and structures in empirical data. It is especially useful in economics because it allows the study of multidimensional phenomena such as irrationality [42]. The essence of correspondence analysis is to reduce the dimension of the studied problem, which consists of recreating the distance between points representing rows and columns of the contingency table in a space with fewer dimensions. Calculations are performed in such a way that the loss of information about the diversity of rows and columns of the contingency table is as small as possible. Contingency tables contain appropriate measures to describe the relationships between rows and columns. The final results of the correspondence analysis are two or three-dimensional charts called biplots, which graphically present the relations of co-occurrence between the studied variables. In this study, row and column profile standardization was chosen to simultaneously analyze points representing row profiles and column profiles. The evaluation of points representing the individual variables, the $\chi^{2}$ metric is used, which is the weighted Euclidean distance. In the correspondence analysis, there is a total inertia that signifies the ratio of the $\chi^{2}$ statistic to the grand total of quantity. This is a measure of the dispersion of row profiles and column profiles around average profiles. Inertia that is close to zero signifies a small dispersion of profiles around the average profile. For example, this situation occurs when all students have received the same exam grade.

On some correspondence maps (Figure 3, Figure 4 and Figure 5) [37], the analysis is conducted both from the perspective of enterprise types and ownership sectors, which indicates the need to introduce a method to increase the clarity of these two variables and their states. The easiest way was to combine the points representing the different types and sectors of business ownership so that they formed triangles. Ownership sectors are indicated by hatched triangles, while full-color triangles represent types of enterprises. The vertices of the triangles have two-part names, with the first segment representing the ownership sector and the second segment representing the type of enterprise. In this way, the mixed sector (S3) forms a hatched triangle with vertices S3FR_1, S3FR_2, and S3FR_3, since it includes all three types of enterprises (i.e., small FR_1, medium FR_2, and large FR_3). Since the same principle applies to the other two ownership sectors, in total, there are nine names for the vertices of these triangles. Following this convention, types of enterprises are identified by the second part of their name. Thus, medium-sized enterprises (FR_2) are represented by a triangle with vertices S1FR_2, S2FR_2, and S3FR_2, as this type includes enterprises belonging to public (S1), private (S2), and mixed (S3) sectors. The same principle applies to the other two types of enterprise. The small enterprise type FR_1 is represented by a yellow triangle, the medium-sized enterprise type FR_2 by a pink triangle, and the large enterprise type FR_3 is represented by a light blue triangle.

## 3. Results

Studies on the innovativeness of Polish industrial processing enterprises in three periods, prosperity (2004–2006), global financial crisis (2008–2010), and recovery (2012–2014) led to many interesting discoveries [35,36,37]. The most important of them include:

(1) demonstration of the significant impact of interactions between the ownership sectors and types of enterprises on research results;
(2) detection of a low level of innovativeness of public enterprises compared to other enterprises from different ownership sectors (i.e., private and mixed);
(3) exemplification of Schumpeter’s creative destruction theory by showing that innovative enterprises are developing in accordance with the Red Queen dynamics;
(4) demonstration of the insensitivity of the effects and goals of innovative activity of companies to cyclical factors (business cycle phases);
(5) confirmation of the occurrence of the Polish Green Island effect as a fact and not government propaganda; and
(6) validation of the hypothesis that the global financial crisis is associated with the turn of the Fifth and Sixth Kondratieff waves.

### 3.1. Significant Impact of Interactions between Ownership Sectors and Types of Enterprises on Research Results

**Figure 1:**
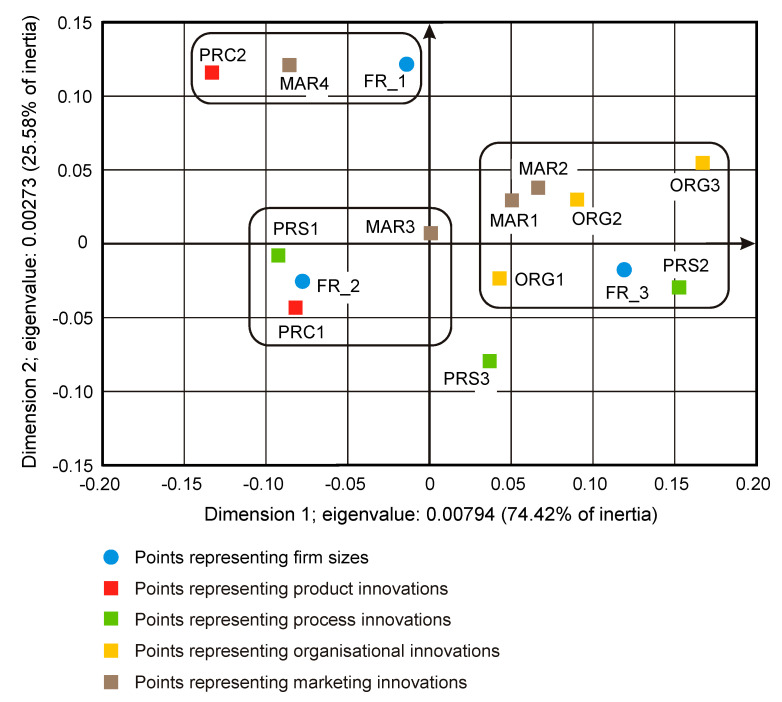
Correspondence map showing the co-occurrence of the types of innovation and the sizes of enterprises in the period 2012–2014 (dimensions 1–2; 100% of total inertia).

**Figure 2:**
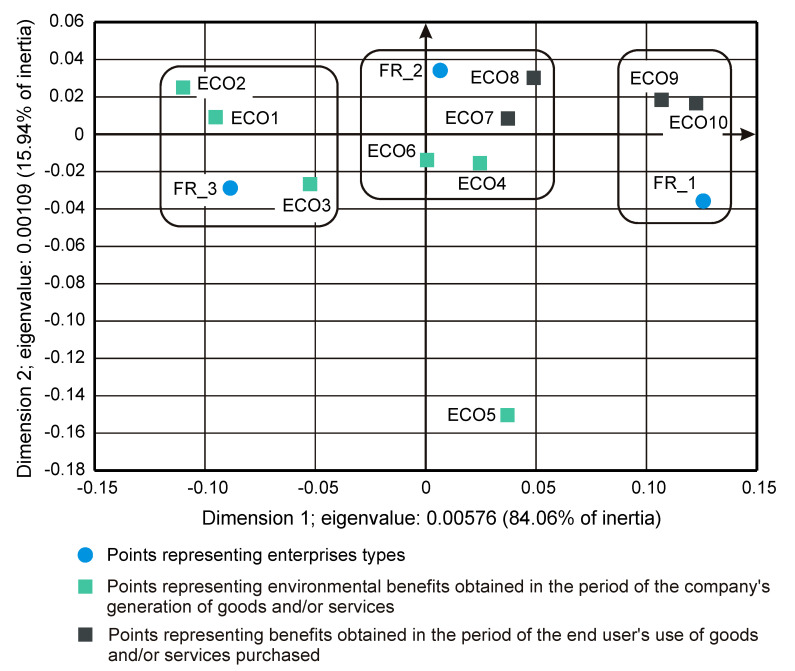
Correspondence map showing the co-occurrence of eco-innovation forms and types of enterprises in the period 2012–2014 (dimensions 1–2; 100% of total inertia).

**Figure 3:**
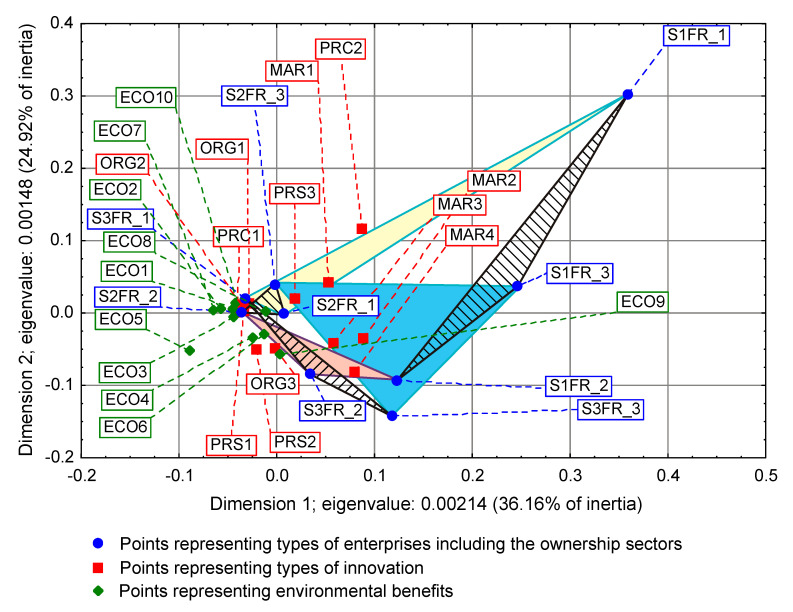
Correspondence map showing the co-occurrence of the types of enterprises including the ownership sectors, the types of innovation, and the environmental benefits in the period 2012–2014 (dimensions 1–2; 61.08% of total inertia).

**Figure 4:**
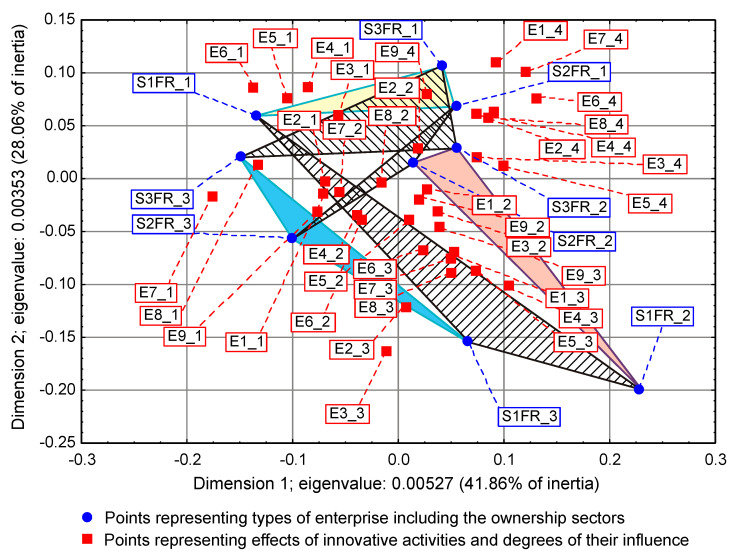
Correspondence map showing the co-occurrence of the types of enterprises including the ownership sectors, the effects of innovative activity, and degrees of their influence on enterprises in period 2004–2006 (dimensions 1–2; 69.92% of total inertia).

**Figure 5:**
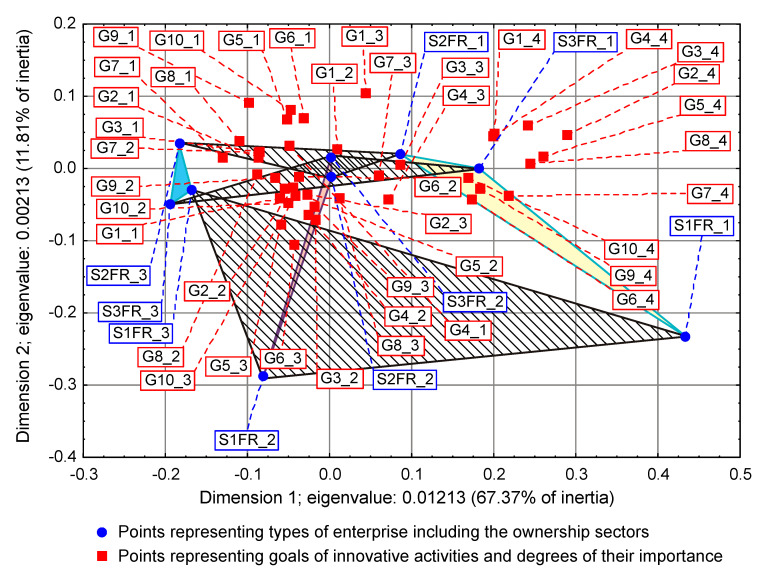
Correspondence map showing the co-occurrence of the types of enterprises including the ownership sectors, the goals of innovative activity, and their degrees of importance for enterprises in period 2008–2010 (dimensions 1–2; 79.18% of total inertia).

The importance of the interrelationships between ownership sectors and types of enterprises for the innovation activity of enterprises, and thus what cybernetic diagrams (3)–(8) show can be demonstrated empirically. During the 2012–2014 survey, it became apparent that the $\chi^{2}$ independence test demonstrates a statistically significant relationship only between the types of enterprises and the twenty-two objectives of innovation, which include both the four basic types of innovation (product, process, organizational, and marketing) as well as eco-innovations (Table 6, Table 7 and Table 8 and Figure 1 and Figure 2) [35,37]. When three ownership sectors are included (public, private, and mixed) in the analysis, this relationship disappears (Table 9 and Figure 3) [37]. In other words, during the recovery period, the goals of innovation activities are independent of the interaction between ownership sectors and types of enterprises. Compared to other periods, the correspondence maps (Figure 1, Figure 2 and Figure 3) showed a significant decrease in average distances between points representing ownership sectors and types of enterprises, and points corresponding to the objectives of innovative activity (Table 10 and Figure 4, Table 11 and Figure 5) [35,37]. The total inertia of the entire industrial processing section has become close to zero (Table 12) [37]. This leads to the conclusion that, in principle, 98.9% of companies achieve their goals of innovative activities, which is all but public sector enterprises (Table 5) [37]. In addition, the result is identical when the goals are limited to four basic types of innovations, and eco-innovations will be considered as supplementary points (Table 13) [37]. The $\chi^{2}$ independence test confirms the cumulative impact of ownership sectors and enterprise types on eco-innovation alone (Table 14) [37].

To draw binding conclusions regarding the calculations made for the years 2012–2014, it is required to compare them with the results obtained for the previous two periods. In the years of prosperity in 2004–2006 and during the global financial crisis in 2008–2010, there was a statistically significant relationship between the combined impact of ownership sectors and types of enterprises, and the effects or objectives of innovative activities (Table 15 and Table 16) [36]. In addition, during the crisis, compared to the previous period of prosperity, the innovative activities of most enterprises increased (Figure 4 and Figure 5) [37], which seems to be a peculiarity, but this can be explained by referring to the impact of secular factors. However, in the third period 2012–2014, this trend continued (Figure 3) [37]. Each company tried to be innovative. Only eco-innovations alone proved to be dependent on the combined impact of ownership and types of enterprises (Table 14) [37], but it should be noted that this was a relatively new type of activity for companies at the time. Generally, it should be noted that the last period was special and ground breaking. Almost all enterprises approached a certain development threshold, some more and some a little less [35,36,37]. The significance of these changes will be explained later.

### 3.2. Low Level of Innovativeness of Public Enterprises

In the three periods examined, public enterprises (S1FR_1, S1FR_2, and S1FR_3) showed significantly less innovative activities than enterprises from other ownership sectors. In the first period (2004–2006), the effects of their innovative activities were weak (Figure 4) [37], and in the next two periods (2008–2010 and 2012–2014), the objectives of innovative activities were not substantially achieved (Figure 3 and Figure 5) [37]. If any effects or goals were achieved, then degrees of influence or importance were low or irrelevant. This means that the innovations introduced by enterprises in the first period had virtually no impact on the activities of companies at the end of 2006 and that the goals achieved in the second period had little effect on innovation activities in the field of product and process innovations. Therefore, if we consider the relationships (3), (5), and (7), then it can be stated that in the case of public enterprises, there were no positive feedbacks. Although there were some differences in individual periods, they did not affect the trend described above. After adopting the prosperity period as a benchmark, it can only be said that in the years of the global financial crisis, the situation of small (S1FR_1) and medium-sized (S1FR_2) public enterprises deteriorated and the situation of large public enterprises (S1FR_3) improved (Figure 4 and Figure 5) [37]. However, during the recovery period, there were no significant changes except that the innovation activity of medium-sized enterprises (S1FR_2) improved, and the innovation activity of the large enterprises (S1FR_3) deteriorated (Figure 3) [37].

In general, the low innovativeness of public enterprises does not seem to be a big economic problem, since the share of the public sector in the entire manufacturing sector was small in the first period, and in the following ones, it showed a decreasing trend. In periods of prosperity, crisis, and recovery, this share was 4.37%, 1.18%, and 1.10%, respectively (Table 3, Table 4 and Table 5) [37]. However, when considering the interactions between ownership sectors and types of enterprises, it cannot be excluded that the public sector may have an adverse effect on enterprises belonging to other ownership sectors. On the other hand, one should also take into account the positive impact of companies from the private and mixed sectors on the public sector. It seems that in the manufacturing sector, there may be some kind of a dynamic balance between ownership and the type (size) of enterprises, which changes in particular phases of the business cycle. Perhaps in this way, the adverse effects of cyclical fluctuations on the innovation activities of enterprises are somewhat neutralized. There may also be impacts from factors operating for even longer periods. Nevertheless, this issue requires further in-depth research, and some related proposals are presented in the next two parts of the article.

### 3.3. The Red Queen Effect

The Red Queen effect is a metaphor derived from Alice’s adventures in Wonderland where this Queen reigned [43]. In the Queen’s land, one would have to run as fast as one could in order to keep in the same place. However, if one would wish to get somewhere else, one would have to run at least twice as fast as that. Indeed, after a long and exhausting run, Alice noticed with amazement that she was in the same place as before. Initially, this hypothesis was used in biology to explain the law of extinction, which states that organisms in any adaptive zone die with a stochastically constant rate [44]. The justification for this principle is that adaptation to certain living conditions of one species may change the selection pressure to other species and lead to positive feedback between species. The properties of communities will then change in a directional manner. This is similar to an arms race, which takes place both between species and within them. Organisms must be in constant motion and adapt to change in order to survive. Thus, existence and survival (i.e., being in the same place) requires constant running. It soon became apparent that the Red Queen hypothesis could also explain many economic phenomena [37].

Schumpeter’s creative destruction theory includes the Red Queen effect. According to Schumpeter, economic development is associated with the growth and collapse of companies and entire branches of industry [9,11]. Enterprises cannot last forever, and the reason for their collapse is almost always the lack of adequate capacity to implement innovation. Each innovation success is rewarded with a bonus in the form of profit, which is inherently temporary and tends to decrease during competition and adaptation processes. Therefore, no enterprise is safe against bankruptcy.

The Red Queen effect applies to public sector enterprises (S1FR_1, S1FR_2, and S1FR_3) as their share in the manufacturing section is constantly decreasing (Table 3, Table 4 and Table 5) [37]. Therefore, they cannot cope with competition from private and mixed sector companies. If proper preventive action is not taken, public enterprises may permanently disappear from the manufacturing section. This can entail not only economic, but also political consequences.

### 3.4. Insensitivity of the Effects and Goals of Innovative Activities of Enterprises to the Business Cycle Phases

The conducted research shows that in each of the examined periods, the vast majority of enterprises achieved the assumed effects and goals of innovative activities, which contributed to increasing their subsequent activities in this field to a high or medium degree. In the years 2004–2006, the innovations introduced by enterprises had a great impact on the activities of enterprises at the end of 2006, and in the years 2008–2010, most of the achieved goals contributed significantly to increasing the activity of enterprises in the field of product and process innovations. Therefore, there were positive feedback loops that show relationships (3) and (5). These phenomena are visible on correspondence maps (Figure 4 and Figure 5) in the form of short distances between points representing ownership sectors and types of enterprises, and points responsible for the effects or objectives of innovative activities and the degrees of their influence or importance [37]. A similar phenomenon could not be found in 2012–2014, but only because the statistical form PNT-02 did not contain such information. There is, however, other indirect evidence that, in the third period, there was indeed positive feedback, illustrated by the relationship (7), between innovations undertaken at different times. This is evidenced by the near zero inertia of the entire industrial processing section (Table 12), which signifies that almost all enterprises achieved the assumed goals of innovative activities (Figure 3) [37].

In general, the innovation activity of enterprises varies depending on the phases of the business cycle, but this is not always as expected. In the first period, points representing ownership sectors and types of enterprises as well as points responsible for the effects of innovative activities and high or medium degrees of their influence formed a joint cluster on the correspondence map (Figure 4) [37]. This signifies that most enterprises achieve the intended effects and that positive feedback occurs in accordance with relationship (3). When the period of prosperity is taken as the basis for comparative analysis, it should be noted that during the global financial crisis, there were changes that consisted in the formation of two separate clusters of points on the biplot (Figure 5) corresponding to this period [37]. In the first cluster, which covered the vast majority of enterprises, the average distances between points representing types and ownership sectors of enterprises and points corresponding to the objectives of innovative activity and high or medium degrees of their importance for innovative activity in the field of product and process innovation during this period decreased noticeably. Thus, during the crisis, there was a positive feedback presented by the relationship (5). The second cluster was very small and was relatively far away from the first. The enterprises in this cluster showed very little innovative activity, and if they were already achieving some goals, their degrees of importance were low or irrelevant. The cluster contained a yellow triangle, so it mainly represented small businesses (S1FR_1, S2FR_1, and S3FR_1). It seems that the crisis has sifted companies, and thus separated companies with high innovation activity from those that were not very innovative. This is paradoxical, as the position of more innovative enterprises improved during the crisis, and the position of less innovative companies deteriorated. This observation can be justified by referring again to the Red Queen effect (i.e., an explanation indicating the selection pressure created during the crisis that the best companies exerted on all others). In the third period, ownership sectors and types of enterprises had no impact on the objectives of their innovation activities, which included both four basic types of innovation (i.e., process, product, organizational, and marketing innovations) as well as eco-innovations (Table 9 and Figure 3) [37]. There was a further decrease in average distances between points representing ownership sectors and types of enterprises and points related to the objectives of innovative activity. This demonstrates that almost all companies achieved their objectives and proves the existence of positive feedback assumed by dependence (7). A large increase in the innovative activity of enterprises in this period indicates that they considered introducing innovations as a necessary condition for their development [37].

The observations indicate the existence of a certain trend, which consists in a steady increase in the innovative activity of most enterprises and appears to be largely independent of the phases of the business cycle. The global financial crisis proved to be only a selection mechanism that separated the group of the best companies from the weakest and at the same time improved the situation of the former and worsened the situation of the latter. The first group includes the vast majority of enterprises from the industrial processing section. This leads to the conclusion that this trend may have more to do with secular factors than with cyclical factors.

Interestingly, the bad situation of public sector enterprises also does not seem to be connected with cyclical factors. In addition, political factors do not seem to be responsible for this state of affairs as political changes can be faster than cyclical changes. The indication of the Red Queen effect as a reason therefore has an additional justification in the form of long-term selection pressure exerted on public sector companies by more innovative enterprises from other ownership sectors. This would prove that selection pressure is not cyclical, but secular, which seems reasonable.

### 3.5. The Effect of the Polish Green Island

The next stage of research was to consider the impact of interactions between ownership sectors and types of companies on innovation barriers and the ability of these barriers to inhibit the innovative activity of enterprises (degrees of their influence in the first period or degrees of importance in the second and third period). The aim was to determine the impact of innovation barriers on the innovation activities of enterprises. In each of the three examined periods, the combined effect of two feedback loops was considered, one of which examined the interdependencies between (a) the interaction of ownership sectors and types of enterprises and (b) the effects or objectives of innovative activities and the degrees of their influence or importance, while the other concerned the interdependencies between (c) cooperation of ownership sectors and types of enterprises and (d) barriers to innovation and the degree of their influence or importance. In other words, a total of two cybernetic schemes were examined in each period: (3) and (4) in the years of prosperity 2004–2006, (5) and (6) during the period of the global financial crisis 2008–2010 as well as (7) and (8) in the years of recovery 2012–2014 [36].

**Figure 6:**
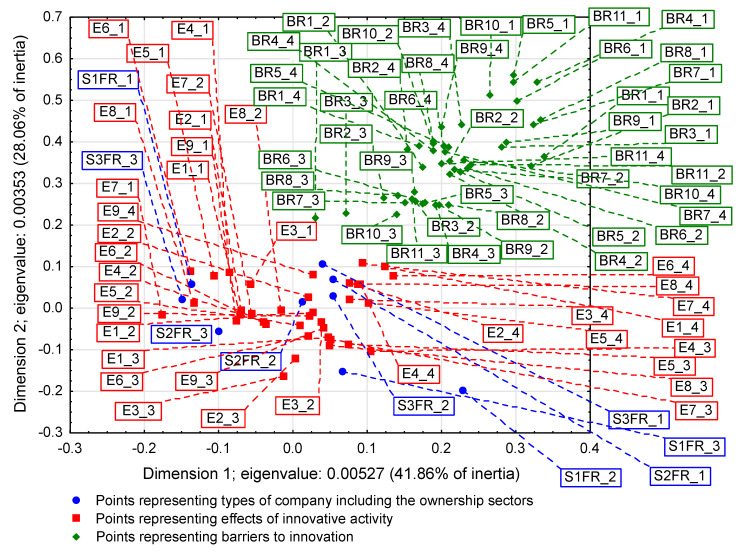
Correspondence map showing the co-occurrence of the types of enterprises including the ownership sectors, the effects of innovative activity, and the barriers to innovation in the period 2004–2006 (dimensions 1–2; 69.92% of total inertia).

The results will be presented chronologically from the first period, which will be the benchmark of comparative analysis. In the years of prosperity, the results of the $\chi^{2}$ independence test confirmed the existence of statistically significant relationships, which concerned both the impact of mutual interactions of ownership sectors and types of enterprises on the effects of innovative activities (Table 15) as well as the impact of mutual interactions of ownership sectors and types of enterprises on barriers to innovation (Table 17) [36]. As a result of using correspondence analysis, a correspondence map (Figure 6) was obtained that captured the co-occurrence of phenomena in more detail [36]. It contained eighty-nine points representing the states of individual variables: nine of them represented ownership sectors and types of enterprises, thirty-six were responsible for the effects of innovative activities of enterprises and degrees of their influence (Table 10), while forty-four were for barriers to innovation and degrees of their influence (Table 18) [36,37]. Points representing barriers to innovation and the degrees of their influence were located at a relatively large distance from a cluster of points consisting of both points representing ownership sectors and types of enterprises as well as points describing the effects of innovation activities and the degree of their influence. Innovation barriers were not overly troublesome for enterprises.

**Figure 7:**
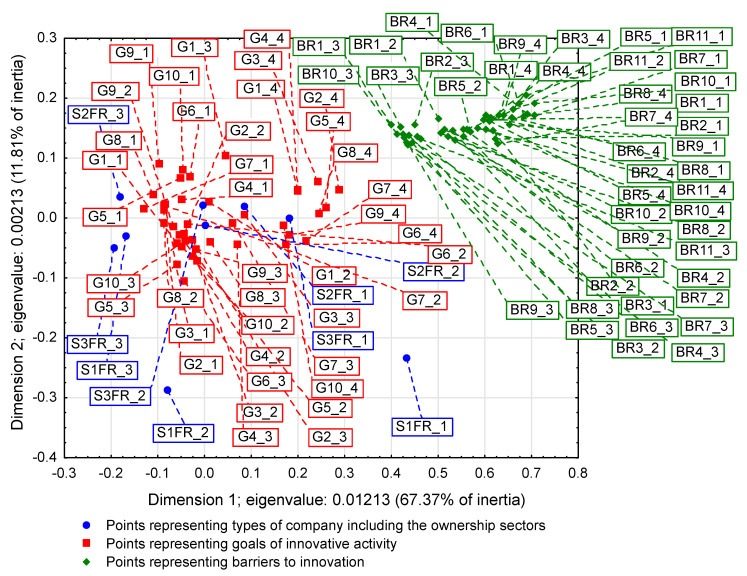
Correspondence map showing the co-occurrence of the types of enterprises including the ownership sectors, the goals of innovative activity, and the barriers to innovation in the period 2008–2010 (dimensions 1–2; 79.18% of total inertia).

During the global financial crisis, the results of applying the $\chi^{2}$ independence test were identical because they showed the dependence of goals of innovation activity and innovation barriers on the combined impact of ownership sectors and types of enterprises (Table 16 and Table 19) [36]. The correspondence map (Figure 7) showed the co-occurrence between ninety-three points representing the states of individual variables, nine of which represented ownership and size of companies, forty for the goals of innovation activities and their degrees of importance (Table 11), and forty-four barriers to innovation and their degrees of importance (Table 18) [36,37]. In comparison to the previous period, it can be observed that the distance between the two clusters of points representing (1) ownership sectors and types of enterprises as well as the objectives of innovation activities and (2) barriers to innovation and their significance levels increased. This demonstrates that during the crisis, the importance of all innovation barriers decreased quite significantly.

**Figure 8:**
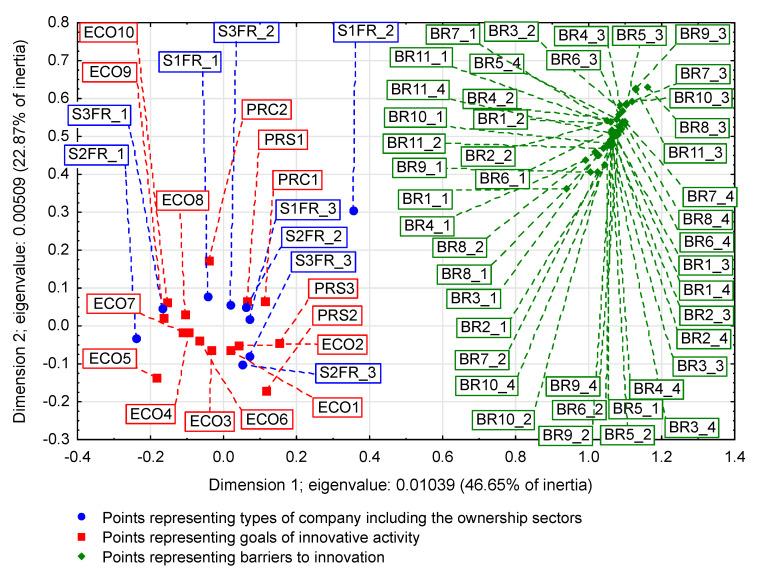
Correspondence map showing the co-occurrence of the types of enterprises including the ownership sectors, the goals of innovative activity, and the barriers to innovation in the period 2012–2014 (dimensions 1–2; 69.52% of total inertia).

In the years of recovery from 2012 to 2014, some stronger alterations could be observed as the $\chi^{2}$ independence test indicated the dependence of the goals of innovative activity on the combined impact of ownership and size of enterprises (Table 20), but did not confirm the existence of the relationship between innovation barriers and the combined impact of ownership and size (Table 21) [36]. The goals of innovative activity (fifteen in total) included product and process innovations as well as eco-innovations (PRC1, PRC2, PRS1–PRS3, ECO1–ECO10). A similarity to the previously obtained result (Table 14) was observed here, which confirmed the importance of eco-innovations for the examined enterprises [37]. Furthermore, it has been found that eco-innovations in Poland are more related to product and process innovations than to organizational and marketing innovations. During this period, the significance of the goals of innovation activities was not considered. Sixty-eight points were included on the correspondence map (Figure 8) depicting the co-occurrence of phenomena, nine of which represented ownership and size, fifteen the objectives of innovative activity (Table 6), and forty-four the barriers to innovation and their degrees of importance (Table 22) [36,37]. Compared to the crisis period, the cluster of points containing ownership and size as well as the objectives of innovative activity is further away from the cluster of points representing barriers to innovation and their degrees of importance. It can be concluded that during the recovery period, nothing prevented the innovative activity of enterprises.

Summing up the research, it can be stated that in the three studied periods, there was a constant tendency to shorten the distance between the points in the two clusters: (1) representing ownership sectors and types of enterprises as well as effects or goals of innovative activity along with degrees of influence or degrees of importance and (2) corresponding only to innovation barriers and degrees their importance. These changes were accompanied by a gradual increase in the distance between these two clusters. It can be observed that these two clusters gradually thickened and at the same time moved away from each other. The trend emerging from these processes showed two simultaneously occurring phenomena: (1) an increase in the innovative activity of enterprises, which is reflected in the implementation of their goals of innovative activities and the appearance of related positive feedback and (2) a gradual decrease in the importance of innovation barriers and the emergence of positive feedbacks related to this process, which led to the almost complete lack of significance of these barriers for enterprises. This long-term trend is independent of the business cycle phases. This signifies that we are dealing with phenomena shaped by pro-developmental secular factors. Most likely, their impact is not limited to the industrial processing section, but applies to the entire economy, which must therefore have a solid, strong foundation for economic growth and development. In order to justify this claim, it is necessary to study the latest economic history of the country.

In 2010, the Polish government presented to the public a map of Europe on which individual countries were attributed the actual economic growth rates they achieved in 2009. Poland had a positive growth rate and was marked in green, while in all surrounding countries, the growth rates were negative, which is why those countries were marked in red. In this way, Poland was presented as the Green Island of economic growth against the background of Europe in crisis [45,46]. In the critical year 2009 for the Polish economy, the growth rate decreased to 1.7%, while the EU average at that time was negative and amounted to –4.2% [47] (p. 91). According to the latest data presented at the Economic Forum in Krynica-Zdrój in 2019, Poland has been recording uninterrupted economic growth since 1992 with an average annual growth rate of over 4%. Over the past 27 years, only Australia has achieved a similar result among the OECD (Organisation for Economic Co-operation and Development) countries. In the years 1990–2018, GNP tripled, and the Polish economy is currently the seventh largest economy in the European Union and the twenty-third in the world [48]. In addition, forecasts show that by 2025, the Polish economy may become one of the strongest engines of growth in Europe and a significant force in the global market [49]. For these reasons, we have called the long-term trend discussed above as the Polish Green Island effect. The map from 2010 was therefore not associated with the government’s propaganda, as was often presented, but showed an actual economic success.

### 3.6. The Global Financial Crisis as the Turn of the Fifth and Sixth Kondratieff Waves

The long-term trends described above apparently lead to some culmination in the period 2012–2014. We observed a decreasing inertia of the industrial processing section, which during the recovery period 2012–2014 became close to zero (Table 12) [37]. This is tantamount to achieving the assumed goals of innovative activity by most enterprises. Moreover, there is a constant decrease in the importance of innovation barriers, until their almost complete disappearance in the third period (Figure 6, Figure 7 and Figure 8) [36]. The second of these phenomena affects the first, which is why they undoubtedly form a certain systematic integrity. It should also be noted that as part of the observed trend, the total impact of ownership sectors and types of enterprises on the innovation activity of companies is significantly reduced (Table 9, Table 15, and Table 16) [36,37]. Therefore, it seems that ownership and size are the factors determining the innovativeness of companies only in relatively short periods appropriate for traditional business cycles. The emergence of this trend can only be explained by secular factors, which indicates the need to interpret the results presented here as part of Kondratieff long-wave theory. Therefore, at least changes in technology and adaptation processes of the Polish economy to the conditions of the world economy should be taken into account. When it comes to technological innovations, it should be emphasized that they are almost always the result of technological revolutions. In this context, it is justified to refer to the modern interpretation of K-waves.

The theory of the Kondratieff cycle was developed and adapted to modern conditions by Šmihula. In the modern age, counted from 1600, he distinguished the following six K-waves [8]:

(1) financial-agricultural revolution (1600–1780; 180);
(2) industrial revolution (1780–1880; 100);
(3) technological revolution (1880–1940; 60);
(4) scientific-technological revolution (1940–1985; 45);
(5) information and telecommunications revolution (1985–2015; 30); and
(6) post-information technological revolution, in other words, the biomedical-hydrogen revolution (2015–2035; 20).

The duration and length of each wave are given in brackets. A characteristic feature of the presented concept is the shortening of the length of each subsequent wave, which is explained by the acceleration of scientific and technological progress. Obviously, the latest wave is predictive, however, it should be noted that its beginning was dated to 2015, so it almost coincided with the period of significant structural changes in the Polish manufacturing industry.

According to Šmihula, the typical end of every K-wave is the economic crisis, which is characterized by stagnation caused by technological stalemate and increased demand for new inventions and innovations [8]. The crisis ending the application phase creates good conditions for the emergence of new inventions, but it takes some time for a new technological revolution to start and technological innovations capable of stimulating investment growth to appear. In his opinion, these changes are practically impossible to be proven by statistical methods due to strong relativism in the assessment of inventions and innovations.

The results presented in this article seem to coincide with the concept of modern K-waves that end and begin with an economic crisis. Considering the two factors mentioned by Kondratieff [7], namely changes in technology and the assimilation of new countries into the world economy, it can be assumed that the transitions occurring in the Polish manufacturing sector are part of a larger whole. If we consider the Fifth Kondratieff wave (i.e., information and telecommunications revolution), it seems that many signs of the end of its application phase can be observed. As a result of continuous improvement of the related innovations, it can be observed that information technology has long become a part of everyday economic life. It is likely that greater profits and revolutionary inventions may appear soon in other industries. There are also many indications that the Sixth Kondratieff wave will be associated with the biomedical-hydrogen revolution. With this approach to the problem, the discovered trend is a fragment of a global phenomenon that is associated with the breakthrough between the Fifth and Sixth Kondratieff waves [37]. The Polish economy is already so integrated with the global economy and included in international supply chains that it can reflect global trends. Therefore, the breakthrough can be determined by statistical methods, however, secular factors have to be considered.

## 4. Discussion

This paper is a summary of the research carried out so far in the area of innovation in Polish manufacturing enterprises [35,36,37]. The obtained results prove that the cybernetic approach is of great importance in researching the innovative activity of enterprises. The ownership sector and the type of enterprise are among the most important factors of innovative activity, however, considering their impact separately may cause that some phenomena remain unrecognized. Examining the mutual influence of these variables has a significant impact on results, as demonstrated by the example of the third period. Focusing only on the types of enterprises, their statistical significant impact on the goals of innovative activity was confirmed, however, after including ownership sectors in the considerations and after taking into account the interactions between ownership and the size of the enterprises, the situation changed radically. This means that the long-term trend referred to in the article could remain undiscovered.

Regarding the poor performance of public enterprises in the field of innovation, it was assumed that one of the reasons may be the political criteria for the selection of managerial staff in these enterprises. Another explanation points to significant pay disparities between employees in the public and private sectors that are working to the disadvantage of the public sector. However, research shows that in terms of innovation, public sector enterprises do not have to perform worse than private ones. An example is Chinese state-owned enterprises, which have gained an advantage over private companies in the field of process innovation. It should not be forgotten, however, that in China after 2000, over 90% of government-owned corporations adopted the Modern Enterprise System, which consisted of implementing corporate or shareholding reforms and adopting a sound corporate structure, as a result of which the boards of shareholders, directors, supervisors, and managers were created in them [34]. Perhaps similar reforms are required by Polish industrial processing enterprises operating in the public sector.

The Red Queen hypothesis indicates that the source public enterprises’ problems may be the strong selection pressure exerted on them by a more innovative environment. The gradual decline in the share of public enterprises in the manufacturing section is worrying, as it may be a source of some political and economic perturbations and have a negative impact on the entire economy. Some enterprises should certainly remain public due to the need to achieve certain social goals.

The insensitivity of the effects and goals of the innovative activity of enterprises to the business cycle phases most likely means that in the total span of the three studied periods, the importance of secular factors was greater than that of business cycles. From this point of view, the global financial crisis should be treated as a phenomenon that strengthened and accelerated the operation of the selection mechanism, as a result of which most innovative enterprises improved their market position. Some less innovative enterprises could also benefit as the Red Queen effect could have forced them to have a reverse reaction in the form of increased innovation efforts. The appearance of positive feedback would mean that the crisis probably accelerated the innovation race, thus improving the situation of most companies in the third period, as evidenced by the near zero inertia of the system under study.

The effect of the Polish Green Island signifies that very good results of the majority of industrial processing enterprises in the field of innovation depend to a small extent on the phases of the business cycle. In the three examined periods, the majority of enterprises did not have problems with achieving the assumed effects or goals of innovative activity, and barriers to innovation gradually disappeared [35,36,37]. This is demonstrated by Poland’s economic successes in the last thirty years, which would not have been possible without the great innovation activity of enterprises. In addition to the strong and sustained economic growth mentioned earlier, there are other important achievements to be mentioned including economic opening to the world, reduction of inflation, and increase in welfare [48]. When the studied periods are examined in a comprehensive way, it can be observed that the Polish economy is more affected by secular trends than by cyclical fluctuations. Entrepreneurs have a good understanding of these phenomena, which indicates the appropriate use of information sources for innovative activities.

Research on innovation has provided convincing statistical evidence to support the claim that the global financial crisis heralded a breakthrough that was the end of the Fifth and the beginning of the Sixth Kondratieff wave. The following two discoveries should be mentioned here: the trend associated with the decreasing inertia of the industrial processing section, which has recently been close to zero, and the essential importance of ownership, as its inclusion in the analysis has completely changed the results. The implementation of the goals set by most enterprises in 2012–2014 may indicate that the application phase of the Fifth K-wave, associated with the information and telecommunications revolution, is slowly coming to an end. The level of competition in the manufacturing industry gradually increased, so in the third period, it could already be close to the maximum. The deadlock can be broken only due to new, breakthrough technological innovations that will open new opportunities for economic growth, profit increase, and competition. Information technology has already become an integral part of everyday economic life and will certainly be further used and refined, but it has apparently already used its potential as a driving force for economic growth and development. Many new inventions have appeared on the horizon that are related to biotechnology, nanotechnology, biomedicine, and hydrogen as the fuel of the future. Soon, they can become the basis of a new technological revolution that will initiate the Sixth K-wave. The results presented in this article are consistent with the forecast of Šmihula [8], which dated the beginning of the biomedical-hydrogen revolution for 2015. Therefore, it is quite possible that we are already living in the Sixth K-wave without knowing anything about it. We are reminded in this respect of Monsieur Jourdain, the hero of a five-act comédie-ballet *The Middle Class Gentleman* written by Molière, who said to the Master of Philosophy [50]: *By my faith! For more than forty years I have been speaking prose without knowing anything about it, and I am much obliged to you for having taught me that*.

## 5. Conclusions

The presented research focused on the impact of ownership sectors and types of enterprises on the innovation activities of companies, considering changes in the economic environment in which these activities happen. In the three examined periods, a gradual increase in innovation activity was found and it was not disturbed by cyclical fluctuations [35,36,37]. All enterprises, apart from public ones, achieved the assumed effects and goals of innovation activities, which contributed to the creation of positive feedback loops leading to further growth of innovation. There was also another phenomenon that is strongly associated with the first, consisting of a gradual decrease in the importance of innovation barriers, until they became practically imperceptible to enterprises in the last of the examined periods. The Red Queen effect indicates strong competition between enterprises in the field of innovation, and data show that most of them have met this challenge. The long-term growth of innovative activity was not even slowed down by the global financial crisis, which became a selection mechanism for enterprises and mobilized them to increase their efforts in the field of innovation. This contributed to even better corporate performance during the recovery period of 2012–2014. These discoveries are irrefutable evidence that the Polish Green Island effect is a real phenomenon, not a government propaganda trick. They also explain the reasons for Poland’s incredible economic success over the past twenty-seven years, which include strong and uninterrupted economic growth, opening of the economy to the world, controlled inflation, and reduction in unemployment.

The cybernetic approach, consisting of considering the combined impact of ownership sectors and types of enterprises on innovation activities, has contributed to the discovery of a long-term trend of a steady decrease in the inertia of the entire industrial processing section. In the last of the examined periods, the inertia was already close to zero. This means that the activity of enterprises was more influenced by secular changes than by cyclical fluctuations. This effect was discovered after including the ownership sectors in the considerations, and it did not occur when only the impact of the size of enterprises on their innovativeness was examined. The secular changes determining the innovative activity of companies were rather external to the Polish economy and were the result of the impact of global trends. It is hard to imagine that the source of these changes might lie in internal conditions because endogenous changes would be too weak compared to exogenous influences. The industrial processing sector simply reflected global trends due to the fact that the Polish economy was already an integral part of the world economy.

Identification of the carriers of impulses from the global economy to the Polish manufacturing sector is a very important issue. This role was undoubtedly played by the ownership because its inclusion in the considerations revealed the trend of decreasing inertia of the examined system. In order to justify this view, it is necessary to establish the ownership structure in the private and mixed sectors, and in particular, to separate the share of foreign ownership in these sectors. Available data show that in 2010, the percentage of foreign capital in basic (share) capital in the Polish processing industry was 47.9%, while in banking, it exceeded as much as 75% [51] (p. 16). This confirms not only that foreign ownership plays the role of a communication channel transferring knowledge from the world economy to the Polish industrial processing section, but also gives an idea of the channel’s high capacity.

The long-term trend of decreasing inertia can serve as evidence that the global financial crisis was associated with the turn of the Fifth and Sixth K-waves (i.e., the transition from the information and telecommunications revolution to the biomedical-hydrogen revolution). Such crisis is a typical phenomenon because it results from the technological deadlock related to the exhaustion of the investment potential of the technology used so far. The technical progress that has taken place in recent years has resulted in many inventions, and these may, in a relatively short time, turn into technological innovations and usher in a new technological revolution. The rate of profit associated with new innovations can be much higher than the one currently provided by information technologies. It is natural, therefore, that during such a crisis, investments decrease and enterprises are looking for new opportunities to develop and overcome competition. When applying this reasoning to the Polish economy, it should be noted that despite its stunning successes as discussed above, the investment rate is decreasing, which is interpreted as a threat to the economic growth in the following years. Of particular concern are the investments of enterprises in machines and devices [48,52]. However, it is possible to look at this problem differently. It cannot be ruled out that entrepreneurs in Poland, knowing about global trends, are refraining from investing in information technologies to take a good position in the upcoming Sixth K-wave. A typical phenomenon is that at the beginning of each wave of technological innovations, there are a lot of relatively small enterprises that use many different technological methods. After some time, as a result of concentration processes, only a few semi-monopolistic enterprises remain on the market, and the number of technological methods decreases to the few most efficient. The current investment problem may therefore be whether to invest in old technologies or new ones.

## Figures and Tables

**Table 1:** The number of enterprises depending on types and sectors of ownership in the three analyzed databases.

Type/ Ownership Sector (Code)	Database								
	2004–2006			2008–2010			2012–2014		
	Small (FR_1)	Medium (FR_2)	Large (FR_3)	Small (FR_1)	Medium (FR_2)	Large (FR_3)	Small (FR_1)	Medium (FR_2)	Large (FR_3)
**Public (S1)**	90	234	120	52	119	73	20	54	39
**Private (S2)**	2222	4725	1110	10,187	4327	1012	2052	1677	906
**Mixed (S3)**	739	690	219	3560	1039	286	1522	3467	507
**Subtotal**	3051	5649	1449	13,799	5485	1371	3594	5198	1452
**Total**	10,149			20,655			10,244		

**Table 2:** Typology of enterprises in the light of European Union standards and the method of coding.

Types of Enterprise/Code	Number of Employees (NE, in Persons)	Annual Turnover (AT, in EUR Million)	Annual Balance Sheet Total (ABS, in EUR Million)
**Micro**	NE < 10	AT ≤ 2	ABS ≤ 2
**Small (FR_1)**	10 ≤ NE < 50	2 < AT ≤ 10	2 < ABS ≤ 10
**Medium (FR_2)**	50 ≤ NE < 250	10 < AT ≤ 50	10 < ABS ≤ 43
**Large (FR_3)**	NE ≥ 250	AT > 50	ABS > 43

**Table 3:** Percentage share of enterprise types (FR) in ownership sectors (S) and percentage share of ownership sectors in enterprise types in the period 2004–2006.

Database 2004–2006 (%)							
Type/Ownership Sector (Codes)	Small (FR_1)		Medium (FR_2)		Large (FR_3)		Subtotal (S)
	Type	Sector	Type	Sector	Type	Sector	
**Public (S1)**	2.95	20.27	4.14	52.70	8.28	27.03	4.37
**Private (S2)**	72.83	27.58	83.64	58.64	76.61	13.78	79.39
**Mixed (S3)**	24.22	44.84	12.22	41.87	15.11	13.29	16.24
**Subtotal (FR)**	30.06		55.66		14.28		Total = 100

**Table 4:** Percentage share of enterprise types (FR) in ownership sectors (S) and percentage share of ownership sectors in enterprise types in the period 2008–2010.

Database 2008–2010 (%)							
Type/Ownership Sector (Codes)	Small (FR_1)		Medium (FR_2)		Large (FR_3)		Subtotal (S)
	Type	Sector	Type	Sector	Type	Sector	
**Public (S1)**	0.38	21.31	2.17	48.77	5.33	29.92	1.18
**Private (S2)**	73.82	65.61	78.89	27.87	73.81	6.52	75.17
**Mixed (S3)**	25.80	72.88	18.94	21.27	20.86	5.85	23.65
**Subtotal (FR)**	66.81		26.55		6.64		Total = 100

**Table 5:** Percentage share of enterprise types (FR) in ownership sectors (S) and percentage share of ownership sectors in enterprise types in the period 2012–2014.

Database 2012–2014 (%)							
Type/Ownership Sector (Codes)	Small (FR_1)		Medium (FR_2)		Large (FR_3)		Subtotal (S)
	Type	Sector	Type	Sector	Type	Sector	
**Public (S1)**	0.56	17.70	1.04	47.79	2.68	34.51	1.10
**Private (S2)**	57.09	44.27	32.26	36.18	62.40	19.55	45.25
**Mixed (S3)**	42.35	27.69	66.70	63.08	34.92	9.23	53.65
**Subtotal (FR)**	35.09		50.74		14.17		Total = 100

**Table 6:** Variables describing the types of innovation, the goals of innovative activity in the years 2012–2014, and coding method.

Types of Innovation		Goals of Innovative Activity	Codes
**Product innovations**		New or significantly improved manufactured goods	PRC1
		New or significantly improved services	PRC2
**Process innovations**		New or significantly improved methods of producing goods and services	PRS1
		New logistic processes	PRS2
		New management processes	PRS3
**Organizational innovations**		New methods under the principles of operation adopted	ORG1
		New methods of distribution of tasks and decision-making powers among employees	ORG2
		New organizational methods in terms of relations with the environment	ORG3
**Marketing innovations**		Significant changes in the design/construction and/or packaging of goods and/or services	MAR1
		New media or product promotion methods	MAR2
		New methods in terms of product distribution or sales channels	MAR3
		New methods of pricing goods and services	MAR4
**Eco-innovations**	**Environmental benefits obtained during the production of products or services**	Reduction of material consumption or water consumption per unit of product	ECO1
		Reduction of energy intensity or carbon dioxide emissions	ECO2
		Reduction of soil, water, air or noise pollutions	ECO3
		Use of materials that are less polluting or less dangerous to the environment	ECO4
		Reduction of the fossil fuels, higher use of energy obtained from renewable sources	ECO5
		Re-use (recycling) of waste, water or materials for personal use or sale	ECO6
	**Environmental benefits obtained during the period of use of the purchased product or use of the service by end users**	Reducing energy consumption or carbon dioxide emissions	ECO7
		Reduction of air, water, soil or noise pollutions	ECO8
		Facilitating the re-use (recycling) of the product after use	ECO9
		Extending the life of products thanks to increased durability and strength	ECO10

**Table 7:** Results of the verification of the null hypothesis regarding the independence of innovation types from enterprise size (2012–2014).

$\mathrm{Pearson}’s\;\chi^{2}$ **Test of Independence**	
**Null hypothesis** $\left(H_{0}\right)$	Types of innovation implemented do not depend on the enterprise size
**Alternative hypothesis ** $\left(H_{1}\right)$	**Types of innovation implemented depend on the enterprise size**
$\chi^{2}$ **statistics value**	117.36
**Critical region**	right-tailed
**Level of Significance** (α)	α=0.05
***p*** **-value** (p)	p=0.0000
**Decision**	Since p<α, $H_{0}$ needs to be rejected in favour of $H_{1}$

**Table 8:** Results of the verification of the null hypothesis regarding the independence of eco-innovation form choice from enterprise types (2012–2014).

$\mathrm{Pearson}’s\;\chi^{2}$ **Test of Independence**	
**Null hypothesis** $\left(H_{0}\right)$	Forms of eco-innovation do not depend on the type of enterprise
**Alternative hypothesis** $\left(H_{1}\right)$	**Forms of eco-innovation depend on the type of enterprise**
$\chi^{2}$ **statistics value**	55.228
**Critical region**	right-tailed
**Level of Significance** (α)	α=0.05
***p*** **-value** (p)	p=0.0001
**Decision**	Since p<α, $H_{0}$ needs to be rejected in favour of $H_{1}$

**Table 9:** List of assumptions and calculations necessary to verify the hypothesis regarding the relationship between the type and ownership sector of an enterprise and the goals of its innovative activity (2012–2014).

$\mathrm{Pearson}’s\;\chi^{2}$ **Test of Independence**	
$\mathrm{Null}\;\mathrm{hypothesis}\;\left(H_{0}\right)\;$	**The type and ownership sector of enterprises have no effect on the goals of innovative activity**
$\mathrm{Alternative}\;\mathrm{hypothesis}\;\left(H_{1}\right)\;$	The type and ownership sector of enterprises have an effect on the goals of innovative activity
$\chi^{2}$ **statistics value**	120.85
**Critical region**	right-tailed
**Level of significance** (α)	α=0.05
***p*-value** (p)	p=0.99759
**Decision**	Since p>α, there are no grounds for rejecting $H_{0}$

**Table 10:** Variables describing the effects of innovative activity of enterprises in 2004–2006, the degrees of influence of innovations introduced by enterprises in 2004–2006 on the activity of enterprises at the end of 2006, and the method of coding.

Effect Type	Effects of Innovative Activity Scale: 1―High; 2―Medium; 3―Low; 4―Irrelevant	Codes	Degree of Influence
**Product effects**	Increase of the product assortment	E1	1, 2, 3, 4
	Entering into new markets or increasing the existing market share	E2	1, 2, 3, 4
	Product quality increase	E3	1, 2, 3, 4
**Process effects**	Improvement in production flexibility	E4	1, 2, 3, 4
	Increase of production capacity	E5	1, 2, 3, 4
	Reduction of labor costs per unit of product	E6	1, 2, 3, 4
	Reduction of consumption of materials and energy per unit of product	E7	1, 2, 3, 4
**Other effects**	Reduction of harmfulness to the environment and improvement of work safety	E8	1, 2, 3, 4
	Compliance with regulations, norms or standards	E9	1, 2, 3, 4

**Table 11:** Variables describing the goals of innovative activity in the years 2008–2010, their degrees of importance for innovative activity of enterprises with regard to product or process innovation in 2008–2010, and the method of coding.

Goals of Innovative Activity Scale: 1―High; 2―Medium; 3―Low; 4―Irrelevant	Codes	Degree of Importance
Increase of the product or service assortment	G1	1, 2, 3, 4
Replacement of obsolete products or processes	G2	1, 2, 3, 4
Entering into new markets or increasing the existing market share	G3	1, 2, 3, 4
Improvement of the quality of products or services	G4	1, 2, 3, 4
Improvement in production flexibility	G5	1, 2, 3, 4
Increase of production capacity	G6	1, 2, 3, 4
Reduction of labor costs per unit of product	G7	1, 2, 3, 4
Reduction of consumption of materials and energy per unit of product	G8	1, 2, 3, 4
Reduction of harmfulness to the environment	G9	1, 2, 3, 4
Improvement of work safety	G10	1, 2, 3, 4

**Table 12:** Total inertia of the Polish industrial processing section.

Total Inertia		
2004–2006	2008–2010	2012–2014
0.0126	0.01801	0.00593

**Table 13:** List of assumptions and calculations necessary to verify the hypothesis regarding the relationship between the type and ownership sector of an enterprise and the goals of its innovative activity with the eco-innovations as supplementary points (2012–2014).

$\mathrm{Pearson}’s\;\chi^{2}$ **Test of Independence**	
$\mathrm{Null}\;\mathrm{hypothesis}\;\left(H_{0}\right)\;$	**The type and ownership sector of enterprises have no effect on the goals of innovative activity, taking into account eco-innovations as supplementary points**
$\mathrm{Alternative}\;\mathrm{hypothesis}\;\left(H_{1}\right)\;$	The type and ownership sector of enterprises have an effect on the goals of innovative activity, taking into account eco-innovations as supplementary points
$\chi^{2}$ **statistics value**	65.248
**Critical region**	right-tailed
**Level of significance** (α)	α=0.05
***p*-value** (p)	p=0.96687
**Decision**	Since p>α, there are no grounds for rejecting $H_{0}$

**Table 14:** List of assumptions and calculations necessary to verify the hypothesis regarding the relationship between the type and ownership sector of an enterprise and the eco-innovations (2012–2014).

$\mathrm{Pearson}’s\;\chi^{2}$ **Test of Independence**	
$\mathrm{Null}\;\mathrm{hypothesis}\;\left(H_{0}\right)\;$	The type and ownership sector of enterprises have no effect on the activity of a firm concerning eco-innovation
$\mathrm{Alternative}\;\mathrm{hypothesis}\;\left(H_{1}\right)\;$	**The type and ownership sector of enterprises have an effect on the activity of a firm concerning eco-innovation**
$\chi^{2}$ **statistics value**	311.44
**Critical region**	right-tailed
**Level of significance** (α)	α=0.05
***p*-value** (p)	p=0.0000
**Decision**	$H_{0}$ hypothesis should be rejected in favour of $H_{1}$

**Table 15:** List of assumptions and calculations necessary to verify the hypothesis about the relationship between the type and sector of enterprise ownership and the effects of its innovative activities (2004–2006).

$\mathrm{Pearson}’s\;\chi^{2}$ **Test of Independence**	
$\mathrm{Null}\;\mathrm{hypothesis}\;\left(H_{0}\right)\;$	The type and ownership sector of the enterprise have no impact on the effects of innovative activity
$\mathrm{Alternative}\;\mathrm{hypothesis}\;\left(H_{1}\right)\;$	**The type and ownership sector of the enterprise have an impact on the effects of innovative activity**
$\chi^{2}$ **statistics value**	426.05
**Critical region**	right-tailed
**Level of significance** (α)	α=0.05
***p*-value** (p)	p=0.0000
**Decision**	$H_{0}$ hypothesis should be rejected in favour of $H_{1}$

**Table 16:** List of assumptions and calculations necessary to verify the hypothesis about the relationship between the type and sector of enterprise ownership and the goals of its innovative activities (2008–2010).

$\mathrm{Pearson}’s\;\chi^{2}$ **Test of Independence**	
$\mathrm{Null}\;\mathrm{hypothesis}\;\left(H_{0}\right)\;$	The type and ownership sector of the enterprise have no impact on the goals of innovative activity
$\mathrm{Alternative}\;\mathrm{hypothesis}\;\left(H_{1}\right)\;$	**The type and ownership sector of the enterprise have an impact on the goals of innovative activity**
$\chi^{2}$ **statistics value**	668.581
**Critical region**	right-tailed
**Level of significance** (α)	α=0.05
***p*-value** (p)	p=0.0000
**Decision**	$H_{0}$ hypothesis should be rejected in favour of $H_{1}$

**Table 17:** List of assumptions and calculations necessary to verify the hypothesis about the relationship between the type and sector of enterprise ownership and innovation barriers (2004–2006).

$\mathrm{Pearson}’s\;\chi^{2}$ **Test of Independence**	
$\mathrm{Null}\;\mathrm{hypothesis}\left(H_{0}\right)\;$	The type and ownership sector of the enterprise have no impact on innovation barriers
$\mathrm{Alternative}\;\mathrm{hypothesis}\;\left(H_{1}\right)\;$	**The type and ownership sector of the enterprise have an impact on innovation barriers**
$\chi^{2}$ **statistics value**	1519.68
**Critical region**	right-tailed
**Level of significance** (α)	α=0.05
***p*-value** (p)	p=0.0000
**Decision**	$H_{0}$ hypothesis should be rejected in favour of $H_{1}$

**Table 18:** Variables describing innovation barriers in the years 2004–2006 and 2008–2010, degrees of their influence on enterprises and coding.

Type of Barrier	Factors Impeding Innovative Activity Scale: 1―High; 2―Medium; 3―Low; 4―Irrelevant	Codes	Degree of Influence
**Economic factors**	Lack of financial resources in your company or in your group of enterprises	BR1	1, 2, 3, 4
	Lack of financial resources from external sources	BR2	1, 2, 3, 4
	Too high costs of innovation	BR3	1, 2, 3, 4
**Knowledge factors**	Lack of qualified staff	BR4	1, 2, 3, 4
	No information about technology	BR5	1, 2, 3, 4
	No information on markets	BR6	1, 2, 3, 4
	Difficulties in finding partners for cooperation in the field of innovative activity	BR7	1, 2, 3, 4
**Market factors**	Market split by dominant enterprises	BR8	1, 2, 3, 4
	Uncertain demand for innovative (new) products	BR9	1, 2, 3, 4
**Other factors**	No need to run innovative activity due to the introduction of innovations in previous years	BR10	1, 2, 3, 4
	No demand for innovation	BR11	1, 2, 3, 4

**Table 19:** List of assumptions and calculations necessary to verify the hypothesis about the relationship between the type and sector of enterprise ownership and innovation barriers (2008–2010).

$\mathrm{Pearson}’s\;\chi^{2}$ **Test of Independence**	
$\mathrm{Null}\;\mathrm{hypothesis}\;\left(H_{0}\right)\;$	The type and ownership sector of the enterprise have no impact on innovation barriers
$\mathrm{Alternative}\;\mathrm{hypothesis}\;\left(H_{1}\right)\;$	**The type and ownership sector of the enterprise have an impact on innovation barriers**
$\chi^{2}$ **statistics value**	3174.84
**Critical region**	right-tailed
**Level of significance** (α)	α=0.05
***p*-value** (p)	p=0.0000
**Decision**	$H_{0}$ hypothesis should be rejected in favour of $H_{1}$

**Table 20:** List of assumptions and calculations necessary to verify the hypothesis regarding the relationship between the type and ownership sector of an enterprise and the goals of its innovative activity in the years 2012–2014 (PRC1, PRC2, PRS1–PRS3, ECO1–ECO10).

$\mathrm{Pearson}’s\;\chi^{2}$ **Test of Independence**	
$\mathrm{Null}\;\mathrm{hypothesis}\;\left(H_{0}\right)\;$	The type and ownership sector of the enterprise have no impact on the goals of innovative activity
$\mathrm{Alternative}\;\mathrm{hypothesis}\;\left(H_{1}\right)\;$	**The type and ownership sector of the enterprise have an impact on the goals of innovative activity**
$\chi^{2}$ **statistics value**	2361.7
**Critical region**	right-tailed
**Level of significance** (α)	α=0.05
***p*** (p)	p=0.0000
**Decision**	$H_{0}$ hypothesis should be rejected in favour of $H_{1}$

**Table 21:** List of assumptions and calculations necessary to verify the hypothesis regarding the relationship between the type and ownership sector of an enterprise and the reasons for the lack of innovation and barriers to innovation (2012–2014).

$\mathrm{Pearson}’s\;\chi^{2}$ **Test of Independence**	
$\mathrm{Null}\;\mathrm{hypothesis}\;\left(H_{0}\right)\;$	**The enterprise type and ownership sector have no impact on the reasons for the lack of innovation and barriers to innovation**
$\mathrm{Alternative}\;\mathrm{hypothesis}\;\left(H_{1}\right)\;$	The type and ownership sector of the enterprise have an impact on the reasons for the lack of innovation and barriers to innovation
$\chi^{2}$ **statistics value**	251.602
**Critical region**	right-tailed
**Level of significance** (α)	α=0.05
***p*** (p)	p=0.9999
**Decision**	Since p>α, there are no grounds for rejecting $H_{0}$

**Table 22:** Variables describing the reasons for the lack of innovation and barriers to innovation in the years 2012–2014, their degrees of importance for enterprises, and the method of coding.

Reasons for a Lack of Innovation	Factors Impeding Innovative Activity Scale: 1―High; 2―Medium; 3―Low; 4―Irrelevant	Codes	Degree of Importance
**No compelling reason for introducing innovation**	Low demand for innovation on market	BR_1	1, 2, 3, 4
	No need to implement innovation due to earlier innovations	BR_2	1, 2, 3, 4
	No need to implement innovation due to low competition on the market	BR_3	1, 2, 3, 4
	Lack of good ideas for innovation	BR_4	1, 2, 3, 4
**The implementation of innovations was considered, but the barriers proved to be too high**	Lack of financing opportunities for innovation from the company’s internal sources	BR_5	1, 2, 3, 4
	Lack of financing for innovation from external sources – loans or funds under private equity financing (including venture capital)	BR_6	1, 2, 3, 4
	No staff with the right skills in your company	BR_7	1, 2, 3, 4
	Difficulties in obtaining public grants or subsidies for innovation	BR_8	1, 2, 3, 4
	No partners to cooperate with	BR_9	1, 2, 3, 4
	Uncertain market demand for your ideas for innovation	BR_10	1, 2, 3, 4
	Too much competition on the market	BR_11	1, 2, 3, 4
